# Assessment of the agreement between the Framingham and DAD risk equations for estimating cardiovascular risk in adult Africans living with HIV infection: a cross-sectional study

**DOI:** 10.1186/s40794-017-0055-z

**Published:** 2017-07-05

**Authors:** Steve Raoul Noumegni, Vicky Jocelyne Moor Ama, Felix K. Assah, Jean Joel Bigna, Jobert Richie Nansseu, Jenny Arielle M. Kameni, Jean-Claude Katte, Mesmin Y. Dehayem, Andre Pascal Kengne, Eugene Sobngwi

**Affiliations:** 1 Faculty of Medicine and Biomedical Sciences, University of Yaoundé 1, PO Box 1364, Yaoundé, Cameroon; 2 Faculty of Medicine, University of Paris Sud XI, 63 Av Gabriel Péri, Le Kremlin Bicêtre, France; 3 Department of Biochemistry and Physiological Sciences, Faculty of Medicine and Biomedical Sciences, University of Yaoundé 1, PO Box 1364, Yaoundé, Cameroon; 4 Laboratory of Biochemistry, University Teaching Hospital, Yaoundé, Cameroon; 5 Department of Public Health, Faculty of Medicine and Biomedical Sciences, University of Yaoundé 1, PO Box 1364, Yaoundé, Cameroon; 6 Department of Epidemiology and Public Health, Centre Pasteur of Cameroon, PO Box 1274, Yaoundé, Cameroon; 7 University of Mons, 20 place du parc PO, 7000 Mons, Belgium; 8 National Obesity Center, Yaoundé Central Hospital, PO Box 87, Yaoundé, Cameroon; 9 Non-Communicable Diseases Research Unit, South African Medical Research Council, Cape Town, 7505 South Africa; 10 Department of Medicine, Groote Schuur Hospital and University of Cape Town, Cape Town, 8000 South Africa; 11 Departement of Internal Medicine and Specialities, Faculty of Medicine and Biomedical Sciences, University of Yaoundé 1, PO Box 1364, Yaoundé, Cameroon; 12 Laboratory of Molecular Medicine and Metabolism, Biotechnology Center, University of Yaoundé 1, Yaoundé, Cameroon

**Keywords:** Cardiovascular risk, HIV, Framingham, DAD

## Abstract

**Background:**

The Absolute cardiovascular disease (CVD) risk evaluation using multivariable CVD risk models is increasingly advocated in people with HIV, in whom existing models remain largely untested. We assessed the agreement between the general population derived Framingham CVD risk equation and the HIV-specific Data collection on Adverse effects of anti-HIV Drugs (DAD) CVD risk equation in HIV-infected adult Cameroonians.

**Methods:**

This cross-sectional study involved 452 HIV infected adults recruited at the HIV day-care unit of the Yaoundé Central Hospital, Cameroon. The 5-year projected CVD risk was estimated for each participant using the DAD and Framingham CVD risk equations. Agreement between estimates from these equations was assessed using the spearman correlation and Cohen’s kappa coefficient.

**Results:**

The mean age of participants (80% females) was 44.4 ± 9.8 years. Most participants (88.5%) were on antiretroviral treatment with 93.3% of them receiving first-line regimen. The most frequent cardiovascular risk factors were abdominal obesity (43.1%) and dyslipidemia (33.8%). The median estimated 5-year CVD risk was 0.6% (25th-75th percentiles: 0.3-1.3) using the DAD equation and 0.7% (0.2-2.0) with the Framingham equation. The Spearman correlation between the two estimates was 0.93 (*p* < 0.001). The kappa statistic was 0.61 (95% confident interval: 0.54-0.67) for the agreement between the two equations in classifying participants across risk categories defined as low, moderate, high and very high.

**Conclusion:**

Most participants had a low-to-moderate estimated CVD risk, with acceptable level of agreement between the general and HIV-specific equations in ranking CVD risk.

## Background

The number of people living with HIV has increased globally, from 28.6 million in 2000 to 36.7 million in 2015, of which 70% live in sub-Saharan Africa [1]. The advent and widespread use of combined antiretroviral therapy (ART) has radically changed the natural history of the HIV infection, with many infected people now living longer and experiencing better quality of life [2]. In fact, from 6.1 million people benefiting from this treatment in 2009, there were 18.2 million people on ART worldwide in June 2016 [1]. However, this improved survival has seen the emergence of non-infectious co-morbidities (NIC) as new threats to the health and life of HIV-infected people. These NIC include cardiovascular disease (CVD), which tends to occur at a much younger age in HIV-infected patients compared to the general population [3, 4]. A study from Norway demonstrated that HIV - infected people have a risk of coronary heart disease twice higher than that of the general population [5]. According to the World Health Organization (WHO) recommendations, prevention of CVD should be based primarily on the assessment of overall cardiovascular risk [6].

Several CVD risk equations have been developed worldwide to assist overall CVD risk assessment [2]. CVD equations applicable to people with HIV include equations developed from the general population such as the Framingham CVD risk equation, but also those developed exclusively from populations with HIV infection such as the Data collection on Adverse effects of anti-HIV Drugs (DAD) equation [7]. Compared with the general CVD risk equation, HIV-specific equations have the advantage of incorporating both traditional CVD factors as well as those specific to people living with HIV infection [7]. To what extent this translates into differential CVD risk estimates when applying the two set of models in HIV-infected people, hasn’t been assessed, particularly in the African setting which is the epicenter of HIV infection. Answering this question is important to inform the uptake of absolute risk model based approach to CVD risk assessment in African people with HIV.

In the current study, we have assessed the agreement between the Framingham general CVD equation and the DAD HIV-specific CVD risk equation, in estimating the 5-year CVD risk in HIV-infected Cameroonians. The hypothesis of our study was that there is a good level of agreement between the general population derived Framingham CVD risk equation and the only available HIV-specific CVD risk equation: the DAD CVD risk equation; therefore allowing us to reliably use each of these CVD risk equation in HIV-infected patients in our milieu.

## Methods

### Study design and setting

This cross-sectional study was conducted from December 2015 to May 2016. Participants were recruited at the HIV day-care unit of the Yaoundé Central Hospital in the Capital City of Cameroon. This is one of the most important HIV clinic in the country. Blood samples were analyzed at the biochemistry laboratory of the Yaoundé University Teaching Hospital, Cameroon. The study was approved by the Cameroon National Ethics Committee for Human Health Research (Ethical approval N° 2015/12/710/CE/CNERSH/SP). Administrative approvals were obtained from authorities of the Yaoundé Central Hospital and the Yaoundé University Teaching Hospital. Participants were informed about the different aspects of the study and were included only after providing a signed informed consent form.

### Participants

All HIV-infected patients reporting for follow-up visits at the HIV clinic of the Yaoundé Central Hospital were approached for consideration to be enrolled in the study. Potentially eligible and consenting participants were included if they were aged 30 to 74 years, had no history of CVD, were not pregnant or breastfeeding for women, were not on lipid-modifying therapy or hormone therapy and were clinically stable.

### Data collection

A standardized pre-tested questionnaire was used for data collection on socio-demographic characteristics, history of HIV infection, and cardiovascular risk factors (hypertension and diabetes) via interview and survey of medical files. Family history of CVD was defined as CVD in a first-degree relative before the age of 55 years for men or 65 years for women [8]. Smoking status was classified as never, former, or current (those who had stopped smoking within the past 12 months were considered current smokers).

All participants underwent a physical examination during which anthropometric parameters and blood pressure were measured. Height was measured to the nearest 0.1 cm, using a stadiometer. Weight was measured to the nearest 0.1 kg, using an electronic scale (CAMRY, Hong Kong, China). Body Mass Index (BMI) was derived as weight (kg)/height*height (m^2^), and participants grouped as underweight (<18.5), normal (18.5–24.9), overweight (25.0–29.9) or obese (≥30.0) [9]. Waist circumference and hip circumference were measured to the nearest 0.1 cm using a measuring tape. Both parameters were used to calculate the waist to hip ratio (WHR). Abdominal obesity was defined according to the IDF criteria (waist circumference > 90 cm for men and >80 cm for women) [10]. Blood pressure was measured using an electronic device (Omron M5-1, Omron Healthcare, Kyoto, Japan). High blood pressure was defined as a systolic blood pressure (SBP) ≥ 140 mmHg and/or a diastolic blood pressure (DBP) ≥ 90 and/or self-reported history of antihypertensive medication [11].

### Blood collection and biochemical assays

Blood was aseptically collected on the day of appointment, after a 12-h overnight fasting, by venipuncture of the brachial vein in a 5 ml sodium fluoride tube and a 5 ml dry tube without a tourniquet or fist clenching. Samples were placed on ice (4 °C) and immediately transported to the biochemistry laboratory where plasma and serum specimen were separated by centrifugation at 3.000 rpm for immediate analyses. We used standard colorimetric procedures to assay fasting plasma glucose (FPG), total cholesterol (TC), triglycerides and high-density lipoproteins cholesterol (HDL-C). Low density lipoproteins cholesterol (LDL-C) was subsequently calculated when the triglyceride (TG) level was equal or below 4 mmol/L using the Friedewald formula and assayed trough standard colorimetric procedures when triglyceride (TG) level was over 4 mmol/L [12].

Dyslipidemia was considered as an elevated level of TC (>6.2 mmol/L) and/or elevated level of LDL-C (>4.1 mmol/L) and/or a low HDL-C (<1.04 mmol/L in men and 1.29 mmol/L in women) and/or a hypertriglyceridemia (≥1.7 mmol/L) [13]. Diabetes was defined by a FPG ≥ 7.0 mmol/L at 2 occasions within at least 48 h, or self-reported history of antidiabetes medications [14].

### Cardiovascular risk assessment

The 5-year CVD risk of each participant was calculated using two CVD risk equations: the CVD risk equation for people living with HIV derived from the DAD cohort, and the general Anderson-Framingham CVD risk equation. The Framingham equation estimates CVD risk through a combination of age, sex, systolic blood pressure, antihypertensive therapy (yes or no), serum TC and HDL-cholesterol values, current smoking status (yes or no) and diabetes (yes or no)) [15]. The DAD CVD risk estimates CVD risk by combining information for age, sex, systolic blood pressure, serum TC and HDL-cholesterol level, diabetes (yes or no), smoking status (yes or no), family history of CVD (yes or no), current use of abacavir, indinavir, or lopinavir (yes or no); and the number of years on indinavir or lopinavir in the equation [7]. Participants were ranked based on their estimated 5-year CVD risk as low (<1%), moderate (1 to 5%), high (5 to 10%), and very high risk (>10%) [7]. In this study, we considered any value ≥5% as high.

### Statistical analysis

Data were analyzed using IBM Statistical Package for Social Sciences (SPSS) version 23.0. (IBM Corporation, United States of America). Results are presented as frequency (percentage) for categorical variables, and mean ± standard deviation (SD) or median (25th-75th percentiles) for continuous variables where applicable. Groups’ comparisons used the Chi-square test, the Student *t*-test or equivalents where appropriate. The continuous agreement between risk estimates from the two equations was assessed using the Spearman correlation coefficient (ρ), while their agreement in raking participants across risk categories was assessed using the Cohen’s Kappa statistic, presented with its 95% confidence interval (CI). A *p*-value <0.05 was used to characterize statistically significant results.

## Results

### Data available

Four hundred and seventy-three people agreed to participate in this study of whom 7 were ineligible: including 4 participants with a history of stroke, and three women who were pregnant. Of the 466 interviewed and examined, 14 did not return for blood sample collection. Therefore, the final analyzed sample included 452 participants.

### General characteristics of the participants

Of the 452 participants included, 361 (79.9%) were women. Their age ranged from 30 to 74 years with a mean of 44.4 years, with men being older than women (47.2 vs. 43.7 years, *p* = 0.001). The majority of participants (84.3%, *n* = 381) were urban dwellers. The diagnosed duration of HIV infection ranged from 2 days to 204 months with a median of 84 months (36–108). Of the 452 participants, 400 (88.5%) were on ART, with ART use being higher in women than in men (90.9% vs. 79.1%, *p* = 0.002). The median duration on ART was 72 months (35–108). The first line ART regimen was the most frequent (93.3%, 373/400), Table 1. Nucleoside reverse transcriptase inhibitors (NRTIs) were present in ART combinations of all participants while non-nucleoside reverse transcriptase inhibitors (NNRTIs) were taken only by patients on first-line treatment. Protease inhibitors were part of ART regimen only in patients on second line treatment (*n* = 27; 6.7%) (Table 1).

**Table 1:** Characteristics of the study population

	Overall (*n* = 452)	Women (*n* = 361)	Men (*n* = 91)	*p*
General characteristics				
Mean age (years)	44.4 ± 9.8	43.7 ± 9.9	47.2 ± 8.8	0.001
Unmarried, n (%)	251 (55.5)	227 (62.9)	24 (26.4)	<0.001
Secondary education or higher, n (%)	289 (63.9)	227 (62.9)	62 (68.1)	0.351
Urban residence, n (%)	381 (84.3)	305 (84.5)	76 (83.5)	0.820
Unemployed, n (%)	219 (48.5)	196 (54.3)	23 (25.3)	<0.001
Family past history of premature CVD, n (%)	46 (10.2)	36 (10.0)	10 (11.0)	0.774
Tobacco use, n (%)	27 (6.0)	10 (2.8)	17 (18.7)	<0.001
HIV infection				
Antiretroviral (ART) use, n (%)	400 (88.5)	328 (90.9)	72 (79.1)	0.002
Median duration ART in months (25th-75th percentile)	72 (35–108)	72.0 (34.0-108.0)	74.0 (36.0 – 106.5)	0.971
First line treatment, n (%)	373/400 (93.3)	304/328 (92.7)	69/72 (95.8)	0.442
NVP (NNRTI)-based ART, n (%)	78/373 (20.9)	67/304 (22.0)	11/69 (15.9)	0.261
EFV (NNRTI)-based ART, n (%)	295/373 (79.1)	237/304 (78.0)	58/69 (84.1)	0.261
PI-based ART, n (%)	27/400 (6.8)	24/328 (7.3)	33/72 (4.2)	0.442
Clinical characteristics				
Mean systolic Blood pressure (mmHg)	123.4 ± 22.5	122.6 ± 23.0	126.5 ± 20.3	0.120
Mean diastolic blood pressure (mmHg)	81.3 ± 13.5	81.3 ± 13.6	81.1 ± 13.2	0.909
Hypertension, n (%)	60 (13.3)	48 (13.3)	12 (13.2)	0.978
Mean body mass index (kg/m^2^)	25.8 ± 5.3	26.2 ± 5.5	24.0 ± 3.9	<0.001
Obesity, n (%)	218 (48.0)	188 (52.1)	29 (31.9)	0.001
Mean waist circumference (cm)	82.1 ± 11.6	82.3 ± 11.9	81.0 ± 10.5	0.303
Abdominal obesity, n (%)	195 (43.1)	185 (51.2)	10 (11.0)	<0.001
Mean hip circumference (cm)	95.1 ± 11.2	96.0 ± 12.0	91.4 ± 8.8	0.001
Mean waist/hip ratio	0.86 ± 0.07	0.88 ± 0.07	0.89 ± 0.06	<0.001
Biological characteristics				
Median CD4 count (cells/mm^3^)	375 (245–532)	375 (245–532)	365 (257–504)	0.926
Mean fasting glycaemia (mmol/L)	5.1 ± 0.9	5.1 ± 0.7	5.2 ± 1.3	0.236
Diabetes, n (%)	9 (2.0)	6 (1.7)	3 (3.3)	0.318
Mean total cholesterol (mmol/L)	4.5 ± 1.0	4.5 ± 1.0	4.3 ± 1.1	0.238
Hypercholesterolaemia, n (%)	26 (5.8)	22 (6.1)	4 (4.4)	0.534
Mean HDL-cholesterol (mmol/L)	1.7 ± 0.6	1.7 ± 0.6	1.5 ± 0.6	0.003
Low HDL, n (%)	106 (23.5)	85 (23.5)	21 (23.1)	0.925
Mean triglycerides (mmol/L)	1.0 ± 0.5	1.0 ± 0.4	1.1 ± 0.6	0.012
Hypertriglyceridaemia, n (%)	35 (7.7)	25 (6.9)	10 (11.0)	0.195
Mean LDLcholesterol (mmol/L)	2.3 ± 0.9	2.3 ± 0.9	2.3 ± 1.0	0.788
High LDL cholesterol, n (%)	17 (3.8)	13 (3.6)	4 (4.4)	0.722
Any dyslipidemia, n (%)	153 (33.8)	122 (33.8)	31 (34.1)	0.961
Median 5-year CVD risk				
DAD equation	0.6% (0.3-1.3)	0.5% (0.3-0.9)	1.4% (0.8-2.7)	<0.001
Framingham equation	0.7% (0.2-2.0)	0.5% (0.2-1.5)	1.8% (0.9-4)	<0.001

Values are count (percentages), mean ± standard deviation or median (25th-75th percentiles)

*ART* antiretroviral therapy, *EFV* Efavirenz, *HDL* high density lipoproteins, *LDL* low density lipoproteins, *NNRTs* non-nucleoside reverse transcriptase inhibitors, *NRTIs* nucleoside reverse transcriptase inhibitors, *NVRP* Nevirapine

### Physical examination and biological profile of participants

Mean values were 25.8 kg/m^2^ for BMI, 82.1 cm for waist circumference, 95.1 cm for hip circumference, 0.86 for waist-to-hip ratio, 124 mmHg for SBP and 81 mmHg for DBP (Table 1). BMI and hip circumference were significantly higher in men compared to women while waist/hip ratio was higher in women. Prevalence rates were 13.5% for hypertension, 48.0% for obesity, and 43.1% for abdominal obesity, with obesity rates always higher in women than in men (both *p* < 0.001). Mean values were 5.1 mmol/L for fasting glycaemia, 4.5 mmol/L for total cholesterol, 1.7 mmol/L for HDL cholesterol and 1.0 mmol/L for triglycerides; with mean HDL cholesterol being higher and mean triglycerides lower in women than in men (both *p* < 0.012). Prevalence rates were 2.0% for diabetes mellitus and 33.8% for any dyslipidemia, with no gender difference (both *p* > 0.318).

HIV serotype data were available for 387 participants. Among these, 383 (99.0%) had HIV-1, three had HIV-2, and one participant had both serotypes. Of the 452 participants, 331 (73.9%) had a recent CD4 count (<6 months) in their files. Values ​​ranged from 2 to 1800 cells/mm^3^ with a median of 375 (245–527) cells/mm^3^.

### Estimated CVD risk and agreement between equations

**Fig. 1:**
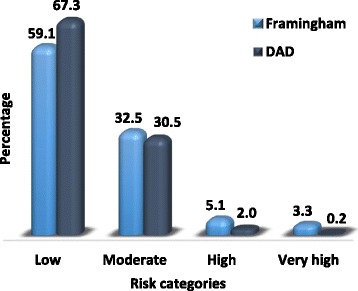
Classification of participants in CVD risk categories by Framingham and DAD equations

The estimated 5- year CVD risk ranged from 0.1 to 13.7%, with a median of 0.6% (0.3-1.3) based on the DAD equation, and from 0.0% to 20.7% with a median of 0.7% (0.2-2.0) based on the Anderson-Framingham equation. Both estimates were higher in men compared to women (both *p* < 0.001). There was a significant positive correlation between estimates from the two equations (ρ =0.93, *p* < 0.001). The classification of participants across risk groups based on the DAD equation was 67.3% (low risk), 30.5% (moderate risk), 2.2% (high risk) and 0.2% (very high risk). Equivalent figures based on the Framingham equation were 59.1, 32.5, 5.1 and 3.3% (Fig. 1). The kappa statistic for the agreement between the two classifications was 0.61 (95% CI: 0.54-0.67; *p* < 0.001) overall, 0.53 (95% CI: 0.39-0.67; *p* < 0.001) for men and 0.59 (95% CI: 0.51-0.67; *p* < 0.001) for women. All participants classified in the high and very high groups by the DAD equation were also classified in the same categories by the Framingham equation (Table 2).

**Table 2:** Cross Classification of participants by Framingham and DAD equations

	CVD risk with Framingham n (%)					
		Low	Moderate	High	Very high	Total
CVD risk with DAD n (%)	Low	259 (57.3)	45 (10.0)	0 (0.0)	(0) 0.0	304 (67.3)
	Moderate	(8) 1.8	102 (22.6)	22 (4.9)	6 (1.3)	138 (30.5)
	High	0 (0.0)	0 (0.0)	1 (0.2)	8 (1.8)	9 (2.0)
	Very high	0 (0.0)	0 (0.0)	0 (0.0)	1 (0.2)	1 (0,2)
	Total	267 (59.1)	147 (32.5)	23 (5.1)	15 (3.3)	452 (100.0)

## Discussion

The mains findings from this study on the agreement between the Anderson-Framingham equation and HIV population specific DAD equation in estimating CVD risk in African adults with HIV infection are the following: 1) levels of classical CVD risk factors were generally lower in this population than in the general population, resulting in low projected 5-year overall CVD risk regardless of the risk estimator used; 2) there was a very good continuous correlation between estimates from the general population based CVD risk equation and the HIV population specific equation, resulting in satisfactory agreement between the two equations in raking participants across risk groups for decision making purposes; 3) although the general equation ranked more participants into the high risk group, this ranking always captured all participants assigned to the high risk group by the HIV-specific equation.

The frequent cardiovascular risk factors in our sample were abdominal obesity and dyslipidemia particularly low HDL-C, in line with a previous report by Edward et al. in Nigeria [16]. Low HDL-C is one of the lipid abnormalities frequently involved in cardiovascular complications among the general population [13]; thus, our results suggest this to be also applicable to HIV-infected patients. General obesity, hypertension, metabolic syndrome and diabetes had respective prevalences of 19.5, 13.3, 11.7 and 2.0%. These prevalences, although lower than those in the general population [17, 18], are similar to those found some years back in South Africa by Julius and co-workers in a population of 304 HIV-infected individuals [19]. Active smoking was relatively low in our study population and concerned mostly men, as previously reported by Menanga and co-workers in Cameroon [20].

Regardless of the estimator used, the 5-year absolute CVD risk was mostly low-to-moderate in our sample, largely in line with existing reports on absolute CVD risk in people with HIV in other settings [2, 21], even when applying the 10-year absolute CVD risk estimates [22–24]. This can be explained at least in part by the relatively young age of participants and high proportion of women, who tend to be at lower CVD risk than men [13].

CVD risk estimates from the Framingham equation were generally higher than those from the DAD equation, although by only a moderate margin; leading however to about three times more participants ranked into the high and very high categories by the Framingham than the DAD equation. Other comparative studies have reported a similar pattern [2, 21]. Despite this trend, estimates from the two equations had an excellent continuous correlation and an average-to-good agreement at ranking participants across risk strata. However, this result was at variance with studies from rural South Africa [21], Brazil [2], and Slovenia [22] where a modest-to-average agreement was observed between DAD and general population derived CVD risk equations. The consistent fact across these studies however is that cross classification of participants by risk categories used 5-year estimated CVD risk categories for the DAD equation and 10-year estimated CVD risk categories for the Framingham and other general CVD risk equations like SCORE or PROCAM. Because change in absolute CVD risk over time is not strictly linear, risk categories from models based on different time-horizons (5-year and 10-year in this case) are unlikely to match perfectly. Learning from our findings, we can therefore speculate that should other studies have used general CVD models for predicting 5-year risk, a better agreement would have been observed.

Moreover, existing studies variably compared the Framingham coronary risk equation based classification against DAD total CVD based classification, which again resulted in low agreement. Indeed, in a study in Slovenia where the 10-year SCORE (a general total CVD equation) was compared against the 5-year DAD, the achieved agreement of 0.53 was closer to the 0.61 observed in our study [22].

Our findings suggest that in routine general adult care settings, the same general population derived risk equation can be used to rank CVD risk regardless of the status for HIV. This will have the undesirable effect of assigning more participants with HIV to the high risk categories. Because people with HIV are likely to be few in such settings, the overall impact will be minimal, while the uptake of absolute risk assessment will be enhanced through the use of a single tool. In HIV clinics however, switching to risk equations specific to people with HIV like the DAD equation could be cost saving. By reclassifying into low risk stratum nearly to 3/4th of those at high risk based on general CVD equation, risk reducing therapies or further investigations could be postponed in a significant number of HIV infected people, protecting them by the same token from the adverse effects of interventions. This thinking however assumes that DAD estimates are better correlated with future CVD risk than general population based equations. In the absence of follow-up with CVD outcomes data collection, we are unable to confirm such a superiority. In the derivation sample of the DAD equation, it performed better than the Framingham equation in prediction incident CVD [7], but this could be due to the so-called ‘home advantage’ or ‘self-fulfilling prophecy’. In a group of 203 HIV-infected people in Spain, the DAD equation was better than Framingham and SCORE at predicting the presence of sub-clinical atherosclerosis [23]. In another study among 83 HIV-infected adults in Slovenia, DAD and SCORE equally ranked most participants with subclinical CVD into the moderate-to-high risk group, compared with Framingham and PROCAM [22].

Our study has some limitations. Participants were all from the same urban referral health facilities, and were not randomly selected, which could limit the generalizability of our findings. Our sample also included a very high proportion of women, which could in some way skew our findings. It is however a general observation that participation of women in surveys tend to be very high across Africa. In the absence of CVD outcome data, we are unable to establish a correlation between true CVD risk and risk estimates from equations developed in non-Africans, applied to our sample. Indeed, difference in background CVD risk and distribution of risk factors across populations [25], may invite the readjustment of those equations in order to obtain accurate estimates of the risk in our population. Strengths of our study include the relatively large sample in comparison with published studies; and unlike those other studies, we compared the DAD and Framingham equation for the same time horizon of predicted probabilities and outcomes. However, further prospective cohort studies with a representative selection of HIV-infected patients are warranted to develop adapted and specific CVD risk equations for this vulnerable population.

## Conclusion

Our findings suggest that in routine clinical settings, application of the same general CVD risk model regardless of the status for HIV is potentially acceptable as it will at most result in more people with HIV classified at high risk, without however unduly ruling out some who could benefit from risk reducing therapies. In typical HIV clinic however, the application of HIV population specific equation could be cost-saving by reclassifying into the lower risk strata, those likely inappropriately ranked as high risk by the general CVD equation. However, the development of locally adapted CVD risk equation including HIV-populations’ specific characteristics, geographical, environmental and ethnic characteristics is warranted.

## Abbreviations

ART: Antiretroviral therapy
BMI: Body mass index
CI: Confident interval
CVD: Cardiovascular diseases
DAD: Data collection on adverse effects of anti-HIV drugs
DBP: Diastolic blood pressure
FPG: Fasting plasma glucose
HDL-C: High density lipoproteins-cholesterol
HIV: Human immunodeficiency virus
IDF: International diabetes federation
LDL-C: Low density lipoproteins-cholesterol
NIC: Non-infectious co-morbidities
NNRTIs: Non-nucleoside reverse transcriptase inhibitors
NRTIs: Nucleoside reverse transcriptase inhibitors
PROCAM: Prospective Cardiovascular Munster
SBP: Systolic blood pressure
SCORE: Systemic coronary risk evaluation
SD: Standard deviation
T-C: Total cholesterol
TG: Triglyceride
WHR: Waist to hip ratio

## References

[1] Fact sheet 2015. [cited 2016 Mar 27]. Available from: http://www.unaids.org/en/resources/campaigns/HowAIDSchangedeverything/factsheet

[2] Nery MW, Martelli CMT, Aparecida Silveira E, de Sousa CA, Falco M de O, de Castro A de CO, et al. Cardiovascular Risk Assessment: A Comparison of the Framingham, PROCAM, and DAD Equations in HIV-Infected Persons. Sci World J. 2013. [cited 2016 Apr 9] Available from: http://www.ncbi.nlm.nih.gov/pmc/articles/PMC3819022/10.1155/2013/969281 PMC3819022 24228022

[3] Triant VA, Lee H, Hadigan C, Grinspoon SK (2007). Increased acute myocardial infarction rates and cardiovascular risk factors among patients with human immunodeficiency virus disease. J Clin Endocrinol Metab.

[4] Boccara F, Meuleman C, Ederhy S, Dufaitre G, Douna F, Lang S, et al. Atteinte cardiovasculaire au cours de l’infection par le VIH. EMC - Cardiol. 2009;4(1):1–7. [cited 2015 Jul 23] Available from: http://www.em-consulte.com/en/article/201119.

[5] Bergersen BM, Sandvik L, Bruun JN, Tonstad S (2004). Elevated Framingham risk score in HIV-positive patients on highly active antiretroviral therapy: results from a Norwegian study of 721 subjects. Eur J Clin Microbiol Infect Dis Off Publ Eur Soc Clin Microbiol.

[6] World Health Organization (WHO) and International Society of… : Journal of Hypertension. LWW. [cited 2015 Jul 23] Available from: http://journals.lww.com/jhypertension/Fulltext/2007/08000/World_Health_Organization__WHO__and_International.9.aspx

[7] Friis-Møller N, Thiébaut R, Reiss P, Weber R, Monforte AD, De Wit S (2010). Predicting the risk of cardiovascular disease in HIV-infected patients: the data collection on adverse effects of anti-HIV drugs study. Eur J Cardiovasc Prev Rehabil Off J Eur Soc Cardiol Work Groups Epidemiol Prev Card Rehabil Exerc Physiol.

[8] Friis-Møller N, Weber R, Reiss P, Thiébaut R, Kirk O, d’Arminio Monforte A (2003). Cardiovascular disease risk factors in HIV patients--association with antiretroviral therapy. Results from the DAD study. AIDS Lond Engl.

[9] Obesity: preventing and managing the global epidemic. Report of a WHO consultation. World Health Organ Tech Rep Ser. 2000;894:i–xii, 1–253. 11234459

[10] IDF Worldwide Definition of the Metabolic Syndrome | International Diabetes Federation. [cited 2016 Feb 28]. Available from: http://www.idf.org/metabolic-syndrome

[11] 1999 World Health Organization-International Society of Hypertension Guidelines for the Management of Hypertension (1999). Guidelines Subcommittee. J Hypertens.

[12] Friedewald WT, Levy RI, Fredrickson DS (1972). Estimation of the concentration of low-density lipoprotein cholesterol in plasma, without use of the preparative ultracentrifuge. Clin Chem.

[13] Expert Panel on Detection, Evaluation, and Treatment of High Blood Cholesterol in Adults. Executive Summary of The Third Report of The National Cholesterol Education Program (NCEP) Expert Panel on Detection, Evaluation, And Treatment of High Blood Cholesterol In Adults (Adult Treatment Panel III). JAMA. 2001;285(19):2486–97. 10.1001/jama.285.19.2486 11368702

[14] WHO | Definition and diagnosis of diabetes mellitus and intermediate hyperglycaemia. WHO. [cited 2016 Feb 28]. Available from: http://www.who.int/diabetes/publications/diagnosis_diabetes2006/en/

[15] Anderson KM, Odell PM, Wilson PW, Kannel WB (1991). Cardiovascular disease risk profiles. Am Heart J.

[16] Edward AO, Oladayo AA, Omolola AS, Adetiloye AA, Adedayo PA (2013). Prevalence of traditional cardiovascular risk factors and evaluation of cardiovascular risk using three risk equations in nigerians living with human immunodeficiency virus. North Am J Med Sci.

[17] Pancha Mbouemboue O, Derew D, Tsougmo JON, Tangyi TM (2016). A community-based assessment of hypertension and some other cardiovascular disease risk factors in Ngaoundéré, Cameroon. Int J Hypertens.

[18] Ama Moor VJ, Ndongo Amougou S, Ombotto S, Ntone F, Wouamba DE, Ngo NB (2017). Dyslipidemia in patients with a cardiovascular risk and disease at the University Teaching Hospital of Yaoundé, Cameroon. Int J Vasc Med.

[19] Julius H, Basu D, Ricci E, Wing J, Basu JK, Pocaterra D (2011). The burden of metabolic diseases amongst HIV positive patients on HAART attending the Johannesburg Hospital. Curr HIV Res.

[20] Menanga AP, Ngomseu CK, Jingi AM, Mfangam BM, Noubiap JJN, Gweth MN (2015). Patterns of cardiovascular disease in a group of HIV-infected adults in Yaoundé, Cameroon. Cardiovasc Diagn Ther.

[21] Mashinya F, Alberts M, Van Geertruyden J-P, Colebunders R (2015). Assessment of cardiovascular risk factors in people with HIV infection treated with ART in rural South Africa: a cross sectional study. AIDS Res Ther.

[22] Pirš M, Jug B, Eržen B, Šabović M, Karner P, Poljak M (2014). Cardiovascular risk assessment in HIV-infected male patients: a comparison of Framingham, SCORE, PROCAM and DAD risk equations. Acta Dermatovenerol Alp Pannonica Adriat.

[23] Serrano-Villar S, Estrada V, Gómez-Garre D, Ávila M, Fuentes-Ferrer M, San RJ (2014). Diagnosis of subclinical atherosclerosis in HIV-infected patients: higher accuracy of the D:a:D risk equation over Framingham and SCORE algorithms. Eur J Prev Cardiol.

[24] Mateen FJ, Kanters S, Kalyesubula R, Mukasa B, Kawuma E, Kengne AP (2013). Hypertension prevalence and Framingham risk score stratification in a large HIV-positive cohort in Uganda. J Hypertens.

[25] GBD 2013 Mortality and Causes of Death Collaborators (2015). Global, regional, and national age-sex specific all-cause and cause-specific mortality for 240 causes of death, 1990–2013: a systematic analysis for the global burden of disease study 2013. Lancet Lond Engl.

